# Mitochondrial genomes of the headstanders *Megaleporinus muyscorum* (Steindachner 1900) and *Megaleporinus obtusidens* (Valenciennes 1837), (Characiformes, Anostomidae)

**DOI:** 10.1080/23802359.2018.1473723

**Published:** 2018-05-23

**Authors:** Melissa Rincón-Sandoval, Ricardo Betancur-R, Javier A. Maldonado-Ocampo

**Affiliations:** a Laboratorio de Ictiología, Unidad de Ecología y Sistemática -UNESIS-, Departamento de Biología, Facultad de Ciencias, Pontificia Universidad Javeriana, Bogotá, Colombia;; b Department of Biology, University of Puerto Rico – Río Piedras, San Juan, Puerto Rico

**Keywords:** Headstanders, mitochondrial genomes, target capture

## Abstract

We report two mitochondrial genomes of headstanders, derived from target capture and Illumina sequencing (HiSeq 2500 PE100). One trans-Andean species *Megaleporinus muyscorum* (mitochondrial consensus genome of 25 individuals) from Colombia and one cis-Andean species *M. obtusidens* from Argentina. Regarding *M. muyscorum*, mitochondrial genome has 13 protein-coding genes, 1 D-loop, 2 ribosomal RNAs, 21 transfer RNAs, and is 14,434 bp in length, for *M. obtusidens* mitochondrial genome has 13 protein-coding genes, 2 ribosomal RNAs, 22 transfer RNAs, and is 15,546 bp in length.

The family Anostomidae is composed by primary freshwater species that occur in South America, commonly known as headstanders that are broadly distributed on both sides of the Andes (Nelson 1994). This family comprises 14 genera and 156 species (Eschmeyer et al. 2018). The species belonging to the genus *Megaleporinus* are used in fisheries and aquaculture and although these species are in a category of least concern according to CITES, it is necessary to follow up the populations and this is one of the reasons why the information of the mitochondrial genome of these species will be of great importance given that have not been published so far.

In this work, 25 individuals in total of *Megaleporinus muyscorum* were collected in field at Atrato, Cauca, Magdalena, San Jorge, and Sinu rivers basins (five individuals per basin) in Colombia and a *M. obtusidens* tissue was from Iguazu (Argentina) belonging to the STRI collection (STRI-2487); for sampling see Appendix S1 (Supporting information). Total DNA was obtained from 20 mg of muscle with the BioSprint 96 from QIAGEN^®^ at The Smithsonian Tropical Research Institute (STRI). Following extraction, we quantified DNA extracts using a Qubit fluorometer (Life Technologies, Inc, Carlsbad, CA) and samples were sent to MYcroarray, Inc (Ann Arbor, MI) for a targeted sequencing approach. Probes were targeted (>200 base pairs) using target capture and Illumina sequencing (HiSeq 2500 PE100). The capture target design is described in detail by Arcila et al. (2017). Although probes were not designed to target mtDNA genes, we tested whether the raw Illumina data contained mtDNA genomes.

We mapped reads from each sample to this most relative available mitochondrial genome of reference *M. elongatus* (GenBank NC034281) using SAMTOOLS (v1.3.1; Li et al. 2009). Then, we winnowed the resulting SAM to only those read alignments hitting the highest-hit references for each locus that were selected for downstream analysis. After that, we removed PCR duplicates converting the SAM to BAM and used the SAMTOOLS rmdup program. The final rmdup.bam file that contained all contigs, was imported to GENEIOUS (v8.1.9; Kearse et al. 2012). Contigs were assembled by mapping sequences against the master reference using the ‘high sensitivity/medium’ algorithm with five iterations and ends were trimmed using defaults. A consensus sequence per each species was extracted from the multiple alignment, and were cleaned posteriorly and visually inspected to detect stop codons. The mtDNA genome was annotated using MitoAnnotator (Iwasaki et al. 2013).

The mitochondrial consensus genome of *M. muyscorum* (Genbank MH286914) is complete, with 13 protein-coding genes, 1 D-loop, 2 ribosomal RNAs, and 21 transfer RNAs (we couldn’t recover through capture 1 tRNA) for a total length of 10,139 bp. For *M. obtusidens* (Genbank MH286915) we couldn’t recover D-loop, this genome has 13 protein-coding genes, 2 ribosomal RNAs, and 22 transfer RNAs for a total length of 14,270 bp. Base compositions are typical for vertebrate mitochondrial genomes with 29% A, 25.8% C, 17.9% G, 27.3% T for *M. muyscorum* and 28.5% A, 27.9% C, 17.5% G, 26.1% T for *M. obtusidens*.

We reconstructed a phylogenetic tree based on the complete mitochondrial genome of 26 individuals belonging to the genus *Megaleporinus*. The Bayesian tree was performed using MrBayes (v3.2.6; Huelsenbeck and Ronquist 2001; Ronquist and Huelsenbeck 2003) for 8 MCMC runs, 10 million of generations, and sampled every 1000. Cis-andean species *M. obtusidens* and *H. malabaricus* are both the sister group of the trans-Andean species; three remarkable cluster are depicted in Figure 1. Atrato, Sinu, and the other one comprise the individuals belonging to the great Magdalena basin.

**Figure 1.**
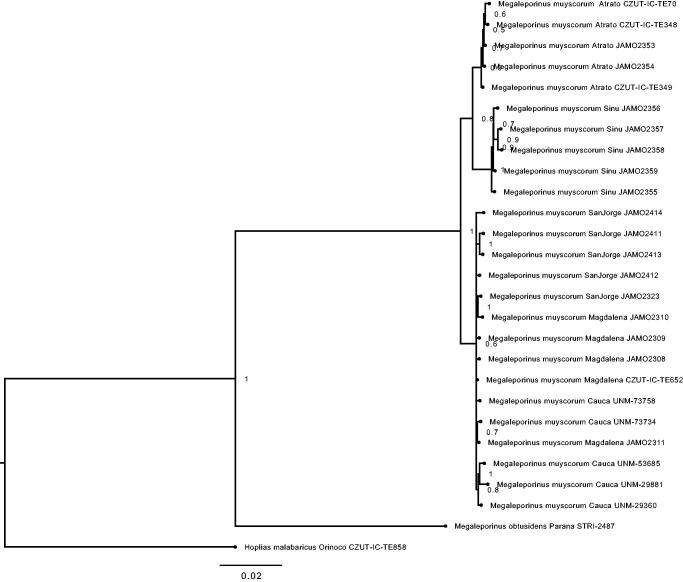
Molecular phylogeny of headstanders Megaleporinus muyscorum and Megaleporinus obtusidens, based on complete mitogenome. Support values at each node are Bayesian posterior probabilities. Branch label include information about sampled basin and tissue availability (JAMO: Pontificia Universidad Javeriana; CZUT-IC-TE: Universidad del Tolima; UNM: Universidad Nacional de Colombia – Medellin; STRI: Smithsonian Tropical Research Institute).

## Supplementary Material

R_AppendixS1.xlsx
